# Detection and Quantification of eDNA-Associated Bacterial Membrane Vesicles by Flow Cytometry

**DOI:** 10.3390/ijms20215307

**Published:** 2019-10-25

**Authors:** Valentina Puca, Eva Ercolino, Christian Celia, Giuseppina Bologna, Luisa Di Marzio, Gabriella Mincione, Marco Marchisio, Sebastiano Miscia, Raffaella Muraro, Paola Lanuti, Rossella Grande

**Affiliations:** 1 Department of Medicine and Aging Science, University “G. d’Annunzio” of Chieti-Pescara, Via dei Vestini, 31, 66100 Chieti, Italy; valentina.puca@unich.it (V.P.); evin30@libero.it (E.E.); giuseppina.bologna@hotmail.it (G.B.); m.marchisio@unich.it (M.M.); s.miscia@unich.it (S.M.); 2 Center for Aging Science and Translational Medicine (CeSI-MeT), Via Luigi Polacchi, 11, 66100 Chieti, Italy; rossella.grande@unich.it; 3 Department of Pharmacy, University “G. d’Annunzio” of Chieti-Pescara, Via dei Vestini, 31, 66100 Chieti, Italy; christian.celia@unich.it (C.C.); luisa.dimarzio@unich.it (L.D.M.); 4 Department of Medical, Oral, and Biotechnological Sciences, University “G. d’Annunzio” of Chieti-Pescara, Via dei Vestini, 31, 66100 Chieti, Italy; gabriella.mincione@unich.it (G.M.); raffaella.muraro@unich.it (R.M.)

**Keywords:** bacterial membrane vesicles, flow cytometry, *Lactobacillus reuteri*, *Helicobacter pylori*, biofilm, extracellular DNA (eDNA), extracellular vesicles

## Abstract

Bacteria generate membrane vesicles, which are structures known as extracellular vesicles (EVs), reported to be involved in different pathogenic mechanisms, as it has been demonstrated that EVs participate in biofilm formation, cell-to-cell communication, bacteria–host interactions, and nutrients supply. EVs deliver nucleic acids, proteins, and polysaccharides. It has been reported that *Helicobacter pylori (H. pylori)* and *Lactobacillus reuteri (L. reuteri)*, of both planktonic and biofilm phenotypes, produce EVs carrying extracellular DNA (eDNA). Here, we used polychromatic flow cytometry (PFC) to identify, enumerate, and characterize EVs as well as the eDNA-delivering EV compartment in the biofilm and planktonic phenotypes of *H.pylori* ATCC 43629 and *L. reuteri* DSM 17938. Biofilm formation was demonstrated and analyzed by fluorescence microscopy, using a classical live/dead staining protocol. The enumeration of EVs and the detection of eDNA-associated EVs were performed by PFC, analyzing both whole samples (cells *plus* vesicles) and EVs isolated by ultracentrifugation confirm EVs isolated by ultracentrifugation. PFC analysis was performed relying on a known-size beaded system and a mix of three different fluorescent tracers. In detail, the whole EV compartment was stained by a lipophilic cationic dye (LCD), which was combined to PKH26 and PicoGreen that selectively stain lipids and DNA, respectively. Fluorescence microscopy results displayed that both *H. pylori* and *L. reuteri* produced well-structured biofilms. PFC data highlighted that, in both detected bacterial species, biofilms produced higher EVs counts when paralleled to the related planktonic phenotypes. Furthermore, the staining with PicoGreen showed that most of the generated vesicles were associated with eDNA. These data suggest that the use of PFC, set according to the parameters here described, allows for the study of the production of eDNA-associated EVs in different microbial species in the same or several phases of growth, thus opening new perspectives in the study of microbial derived EVs in clinical samples.

## 1. Introduction

Bacterial Membrane Vesicles, which respond to the extracellular vesicles (EVs) definition criteria, are membrane structures, produced by Gram-negative, Gram-positive and Mycobacteria microorganisms during their growth [1,2]. These elements are spherical in shape, with an average size in the range from 20 to 500 nm and deliver several components, such as proteins, nucleic acids, and phospholipids [2]. It has been reported that EVs are involved in a number of physiological and pathological mechanisms, including biofilm formation, inter-cellular cross-talk, as well as bacteria–host and bacterium–phage interactions [3]. Many studies support the hypothesis that the generation of EVs is a controlled and regulated process [4], although Turnbull et al. demonstrated that the EVs’ biogenesis is associated with a rearrangement of the bacterial membranes, derived from lysed cells, which incorporate soluble molecules [4,5]. It is also known that EVs fuse with the outer membrane of Gram-negative cells, while they are not able to interact with Gram-positive bacteria. It was demonstrated that EVs stemming from *Pseudomonas aeruginosa* are able to interact with *Shigella flexneri* pouring their content into the periplasm of eukaryotic cells; conversely, EVs derived from *P. aeruginosa* adhere to the cell wall of *Staphylococcus aureus* [6,7]. The EVs composition and content are strictly dependent on the environmental conditions where they are generated, as well as on the bacterial species from which they are produced and on their growth phase [4]. For example, iron-limiting conditions increase the EVs production from *Helicobacter pylori*, *Haemophilus influenzae*, *Escherichia coli*, and *Vibrio cholera* [8,9]; the oxygen availability affects the generation of EVs [4] and the antibiotic treatment (i.e., polymyxin B or colistin) promotes an increase of EVs production that confers protection to the bacterial cells, thus allowing their survival [10]. A previous study, performed on *Stenotrophomonas maltophilia*, demonstrated that the imipenem treatment increases the production of EVs delivering β-lactamases [11].

Modifications in terms of EVs production and content might also be associated with the growth phase, as well as with the bacterial phenotype (biofilm or planktonic) and the media composition. Park et al. demonstrated that EVs associated to the *P. aeruginosa* biofilm phenotype showed a unique proteome profile, with respect to the one carried by EVs stemming from its planktonic counterpart [12].

*Helicobacter pylori* ATCC 43629 and *Lactobacillus reuteri* DSM 17938 are biofilm-producing strains [13,14], and both the planktonic and the biofilm phenotypes generate EVs (pEVs and bEVs, respectively) carrying extracellular DNA (eDNA). [15,16]. In particular, *H. pylori*-derived bEVs seemed to “bridge” bacterial cells, thus playing a possible structural role; however, the fact that eDNA are carried by EVs, may protect the eDNA from the enzymatic activity of DNase I in breaking up the biofilm. It cannot be dismissed that EVs produced from the biofilm phenotype may represent a shuttle for eDNA transport.

For all of these reasons, we aimed to optimize a simplified Polychromatic Flow Cytometry (PFC) method for a fast identification, enumeration and characterization of bacterial derived EVs, as well as for the analysis of their eDNA-associated subtypes, produced by the planktonic and the biofilm phenotypes of a Gram-negative (*H. pylori* ATCC 43629) and a Gram-positive (*L. reuteri* DSM 17938) bacterium. We demonstrated that PFC can be used as a tool to study the modifications of EVs production under different environmental conditions, as well as for the detection and enumeration of bacterial derived EVs, and in particular eDNA-associated EVs, in clinical samples.

## 2. Results

### 2.1. Evaluation of H. Pylori and L. Reuteri Biofilm Formation

**Figure 1:**
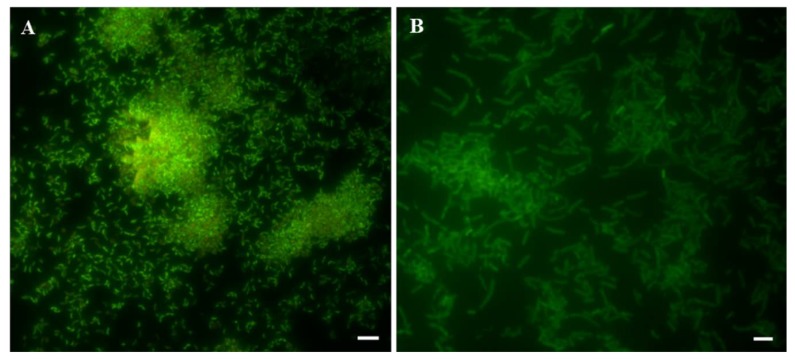
Microscopy representative images of biofilms developed by *Helicobacter pylori* ATCC 43629 after 48 h of incubation (**A**) and *Lactobacillus reuteri* DSM 17938 after 24 h of incubation (**B**). The biofilms were stained with Syto 9 (green fluorescence). Scale bar = 5 μm.

As already suggested [17,18,19,20], we used fluorescence microscopy in order to demonstrate the presence of a well-structured biofilm, developed from both analyzed bacterial strains (Figure 1).

In detail, Figure 1A shows the fluorescence microscopy analysis of the development of a well-structured and mature biofilm, after 2 days. As shown, it is formed by several single cell aggregates of *H. pylori*, which were also heterogeneously interspersed. Many *H. pylori* single cells appeared to be attached to the interstitial channel surface. On the other hand, *L. reuteri* produced a biofilm characterized by a monolayer of cells, which covers the plate surface homogeneously (Figure 1B). The differences in terms of phenotypical biofilm characteristics evidenced when the two microorganisms were paralleled, confirmed previously reported data [14,16,21].

### 2.2. Flow Cytometry Detection and Quantification of Total and eDNA-Associated EVs Generated by H. Pylori and L. Reuteri

**Figure 2:**
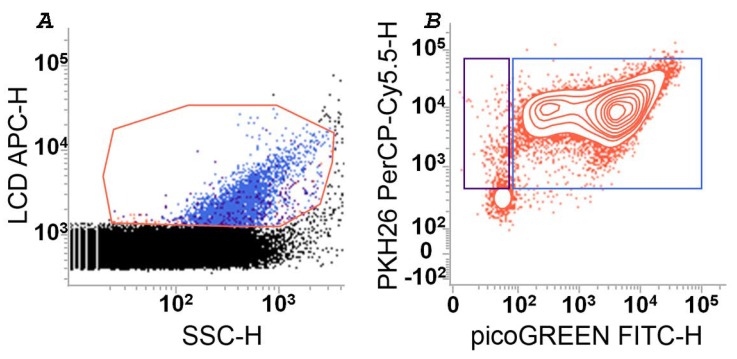
Strategy for flow cytometry detection and enumeration of extracellular vesicles (EVs) produced by *H. pylori* ATCC 43629 and *L. reuteri* DSM 17938. (**A**) EVs were identified on a Lipophilic Cationic Dye-Height (LCD-H)/Side Scatter (SSC)-H dot/plot, as events resulting positive to LCD, having average sizes in the range 160–900 nm (as previously measured by using Mega Mix Plus SSC beads that are used as control). (**B**) EVs were reported on a PKH-26 PerCP-H/pico-Green FITC-H contour-plot and events resulting positive for PKH26 and PicoGreen were defined as “eDNA-associated EVs”. Dot- and contour-plots are representative of at least three independent experiments. The experiments were carried out on whole samples (bacterial cells plus the EVs) and on EVs isolated from the planktonic and biofilm phenotypes.

**Figure 3:**
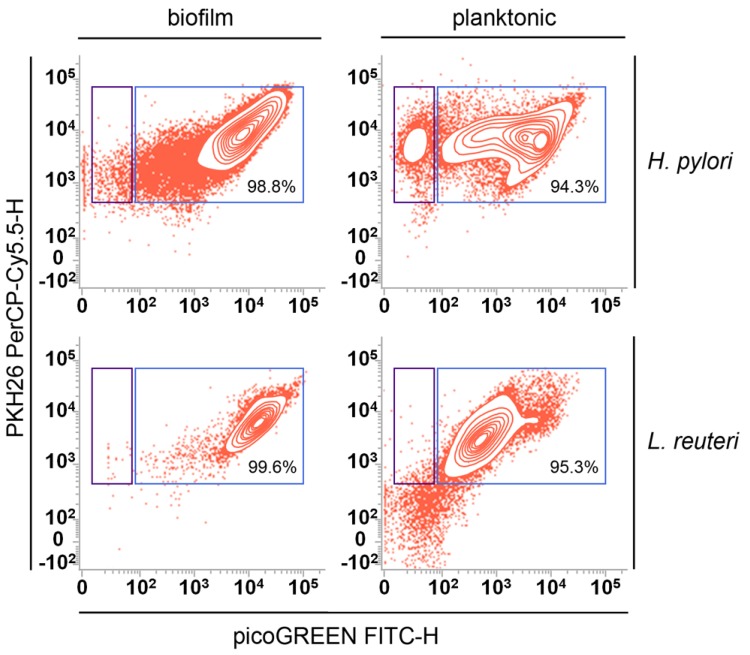
Detection and quantification of vesicles generated by *H. pylori* ATCC 43629 and *L. reuteri* DSM 17938. EVs, identified as described in Figure 2A, were represented for biofilm (bEVs) and planktonic (pEVs) samples on the respective PKH-26 PerCP-H/Pico-Green FITC-H contour-plot, both for *H. pylori* ATCC 43629 and *L. reuteri* DSM 17938. Events resulting positive for PKH26 and PicoGreen were defined as “eDNA-associated EVs”. Contour-plots are representative of at least three independent experiments. The experiments were carried out on whole samples (bacterial cells plus EVs) and on EVs isolated from the planktonic and biofilm phenotypes.

The detection and quantification of EVs isolated by planktonic and biofilm phenotypes of *H. pylori* and *L. reuteri* were carried out on whole samples (cells plus EVs) and isolated EVs. As we have recently demonstrated for EVs derived from eukaryotic cells, the simplified flow cytometry method for EV detection, optimized in our laboratories, was fast and reproducible [22,23,24]. The application of such a method to the microbiological field, allowed us to demonstrate the possibility to analyze EVs displaying an average size ranging from 100 to 200 nm in all analyzed samples. Figure 2 shows the gating strategy used to identify EVs and their sub-populations. As already reported [23], EVs were detected on the basis of their scattered properties and their positivity to LCD (Figure 2A), which stains membrane vesicles, as we have recently published [22,23,24]. EVs were then analyzed for their positivity to PHK26 (which generally stains cell membranes) and PicoGreen (Figure 2B) which is able to identify dsDNA. Therefore, eDNA carrying EVs, both stemming from the biofilm (bEVs) and from the planktonic (pEVs) phenotypes, were identified as PKH26+/PicoGreen+ EVs (Figure 2B). The gates were established as recommended, on the basis of the respective fluorescence minus one controls (not shown). As shown, a large proportion of EVs (>80% of the whole EV population) stained positive for the PicoGreen tracer. Therefore, the resulting data associated with eDNA were highly reproducible, as also demonstrated by Figure 3, where the same analyses, performed both on bEVs and pEVs from *H. pylori* and *L. reuteri*, are displayed.

**Figure 4:**
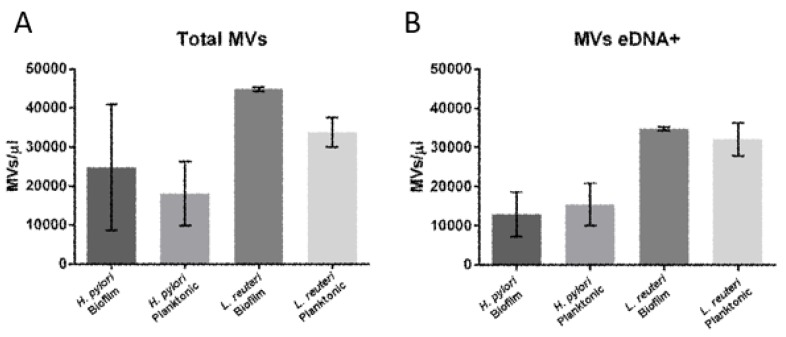
Numbers of EVs. Box plots represent absolute numbers of total EVs (**A**) and MVs containing eDNA (**B**) isolated from the planktonic and the biofilm phenotypes of *H. pylori* and *L. reuteri*. (**A**) Figures are representative of three independent experiments.

Figure 4 shows a greater production of bEVs compared to pEVs for both microorganisms, and the relative concentrations were obtained by flow cytometry volumetric counts: 24,769 bEVs/µL vs 18,048 pEVs/µL in *H. pylori* and 44,887 bEVs/µL vs 33,835 pEVs/µL in *L. reuteri*. In particular, within the EV populations produced by the two detected phenotypes, a higher number of EVs carrying eDNA (average values: 12,968 bEVs/ µL and 15,498 pEVs/ µL for *H. pylori*, and 34,799 bEVs/ µL and 32,056pMVs/ µL for *L. reuteri*) were detected in bEVs. The diameters of the EVs population detected here were overlapping the ones previously measured by Transmission Electron Microscopy [15,16].

### 2.3. Dynamic Light Scattering (DLS) Analysis

Planktonic and biofilm phenotypes of *H. pylori* containing a mixture of bacteria and EVs, produced from their relative strains were analyzed by DLS (Figure S1a,b). In particular, planktonic samples of *H. pylori* showed three different peaks (Figure S1a), measuring: 3.97 nm (Peak 1; 5.4%), 27.88 nm (Peak 2; 15%), and 1783.3 nm (Peak 3; 79.6%). The presence of three different peaks demonstrated that the particles were broadly distributed and heterogeneous samples were obtained before the isolation from bacterial strains. These data were further supported by the respective PDI value (0.81, S.D. 0.3; Table 1). Similar results were obtained for *H. pylori* biofilm samples. Again, three peaks were obtained by the DLS analysis (Figure S1b), and they measured: 2.01 nm (Peak 1; 6.6%), 62.7 nm (Peak 2; 15%), and 332.9 nm (Peak 3; 77.4%). These three different peaks showed that the samples were broadly size distributed, as further supported by the respective PDI value (0.85, S.D. ± 0.1; Table 1).

Purification of EVs from the two bacterial species allowed the collection of more homogeneous samples and the separation of membrane vesicles from bacterial cells. However, some differences occurred between the planktonic and biofilm phenotypes of *H. pylori* and *L. reuteri*. In fact, EVs stemming from the planktonic phenotype (pEVs) of *H. pylori* have an average size of 142.8 nm (S.D. ± 2.4) (Figure S1c) and a size distribution (PDI) of 0.22 (S.D. ± 0.04; Table 1). Conversely, EVs derived from the biofilm phenotype (bEVs) were broadly distributed, and their dimensions ranged from 32.77 to 174.8 nm, with an average size of 1740.4 nm (S.D. ± 1724.97). bMVs of *H. pylori* showed four different peaks (Figure S1d), and the most abundant population of particles had an average size of 123.3 nm (79.6% of particle populations in terms of intensity percentage). The broad distribution of the bEVs of *H. pylori* was further supported by the respective PDI value (0.77, (S.D. ± 0.25; Table 1).

Opposite results were obtained for *L. reuteri* (Figure S2). Planktonic and biofilm forms of *L. reuteri* collected from the bacteria culture before their purification showed narrow size-distributed particles with PDI values below 0.3 (Table 1), in particular, pEVs of *L. reuteri* had a PDI value of 0.26 (S.D. ± 0.02), while bEVs had a PDI value of 0.21 (S.D. ± 0.06). The planktonic phenotype displayed two peaks at 2753 nm (Peak 1; 84.87%), and 3250 nm (Peak 2; 15.13%) (Figure S2a). Conversely, the biofilm phenotype displayed a single peak at 3705 nm (Figure S2b). pEVs and bEVs produced by *L. reuteri* had a broad size distribution with PDI values of 0.61 and 0.75, respectively (Table 1). pEVs had average sizes ranging from 130 to 5400 nm, while bEVs had dipslayed average sizes ranging from 12.25 nm to 5600 nm (Figure S2c). pEVs showed five different peaks at 130.7 nm (Peak 1; 58.9%), 141.7 nm (Peak 2; 60.7%), 388.11 (Peak 3; 29%), 955.4 (Peak 3; 0.2%), 5560 nm (Peak 5; 0.1%). DLS analysis demonstrated that these particles were broadly distributed and the resulting data were in agreement with those obtained for PDI values (Table 1).

The *Z*-potential and electrophoretic mobility values showed negative values for planktonic and biofilm phenotypes of *H. pylori* and *L. reuteri* which were not isolated from bacteria, as well as their EV counterpart (Table 1). These data were consistent with the net negative charge of the cell wall of bacteria. However, the *Z*-potential values were more negative for both pEVs and bEVs than the planktonic and biofilm of mixed samples before EV isolation from *H. pylori* and *L. reuteri* (Table 1).

## 3. Discussion

Bacterial Membrane vesicles (MVs) or Extracellular Vesicles (EVs) are lipoproteic structures, produced by the microorganisms during their growth [2,25]. The number of vesicles could depend on both the bacterial species and growth conditions. Therefore, the evaluation of the number and the content of membrane vesicles, represent a relevant goal to be achieved for the reproducibility of the related scientific data. Indeed, there are several inconsistences in the methods used for the quantification of membrane vesicles, produced in different growth or environmental conditions [4]. The most commonly used methods for the quantification of membrane vesicles are the Nanoparticle Tracking Analysis (NTA), Fluorescence Activated Cell Sorting (FACS), Confocal Laser Scanning (CLSM) or Transmission Electron Microscopy (TEM), the quantification of proteins by SDS-PAGE and densitometry or by Bradford or bicinchoninic-acid assay as well as the quantification of lipids by using the measurement of the absorbance or lipid probes [4,11], [26,27,28,29,30]. The abovementioned methods are characterized both by strengths and limitations, because of the EVs size and the capability to differentiate EVs from non-EV particles (i.e., debris, protein complexes) [4].

As previously shown by Orench–Rivera and Kuehn, the lack of a universally recognized method for the quantification and evaluation of EVs stemming from different microbial species and grown in different growth conditions, represents a goal to be achieved [4]. On the other hand, flow cytometry represents the most promising method for EVs studies. However, even if a relevant number of flow cytometry protocols have been proposed to analyze EVs, experimental data showed a relevant variability in terms of results [31]. In such a context, we have recently optimized and patented a flow cytometry method for the analysis of EVs on whole samples, avoiding the artifact generated by any enrichment procedure (i.e., sample centrifugation) that can artificially generate or disrupt EVs or induce their fusion [24,32].

The abovementioned flow cytometry method recently optimized for eukaryotic derived vesicles [24,32], has been here applied, for the first time, in the microbiological field. It allowed the detection of the smallest particles, as well as a high level of reproducibility.

Therefore, the method here described is highly promising, allowing the detection, quantification, identification, and even the separation (by fluorescence-activated cell sorting) of the eDNA-containing EVs generated from the planktonic and biofilm phenotypes by both Gram-negative and Gram-positive bacteria. This method, optimized in our laboratories (patent code: 102018000003981), is based on the use of a lipophilic cationic dye (LCD) which is able to stain the whole EV compartment and also uses a known size beaded system and two fluorescent dyes, PKH26 and PicoGreen dyes, that selectively stain lipids and dsDNA, respectively.

These parameters have been applied on both whole samples (cells plus membrane vesicles) and isolated vesicles. This method carried out on whole samples allowed us to avoid the underestimation of EV counts. Interestingly, most of the EVs produced from both microorganisms were associated with eDNA in both planktonic and biofilm phenotypes, suggesting that eDNA-associated EVs may play a significant role. The use of PFC, set up according to the here-described parameters, allows a number of applications in the microbiological field, such as the quantification of EVs generated by different bacterial species grown in the same condition, or the quantification of EVs of the same bacterial species grown in different environmental conditions (i.e., sub-inhibitory drug concentrations and variations of temperature or pH). The detection of eDNA-associated EVs represents valuable information to better understand the role of eDNA delivered by these structures. Finally, the possibility offered by the fluorescence-activated cell sorter to purify the eDNA-associated EVs gives the chance to add a defined amount of eDNA-associated EVs to bacterial or eukaryotic cells, in order to study possible eDNA effects.

DLS analysis is commonly used to measure the average sizes and size distribution of colloidal nanoparticles, particularly liposomes [33,34] and polymeric micro- [35] and nano- carriers [36]. Basically, they can have a narrow or broad size distribution based on their lipid or polymeric composition that is similar to biological membranes [37] or metabolites obtained from the physiologic degradation of carbohydrates such as lactide and glycolide compounds [38]. DLS analysis uses Smoluchowski equation and Mie theory to calculate the main parameters of nanoparticles [39] without differentiating between natural and synthetic samples. In fact, extracellular vesicles, viruses, bacteria, dust particles, liposomes and polymeric nanoparticles, having spherical or spherical-like shape, are analyzed like particles and the analysis is not based on their biological origin. Based on this evidence, DLS analysis of *H. pylori* and *L. reuteri* showed that pEVs and bEVs had different peaks widths and a multilayer form. Peaks were differently distributed in planktonic and biofilm samples, and the intensity percentage of the peaks as well as the cumulative distribution of particles depended on the bacterial strains. This was true and might be strictly related to the lipid and protein compositions of *H. pylori* and *L. reuteri*, as previously reported [15,16]. The peak distribution of pEVs and bEVs of *H. pylori* and *L. reuteri* might depend on the first and second order diffraction of lamellar bilayers and its related modification of asymmetric structure as previously reported [15,16], and further demonstrated for various bacterial strains [40]. These properties were also related to the temperature-dependent transition from the liquid to the crystalline phase of lipids forming EVs as previously reported for lipid nanoparticles [41]. The transition from the crystalline to the liquid phase of lipid membranes can increase the fluidity of EVs, thus allowing the fusion between vesicles and their relative increasing of average sizes and sized distribution [40]. The net negative charge of pEVs and bEVs was consistent with the cell wall charge of bacteria, as previously reported [15]. Moreover, differences of Z-potential as absolute values, occurred between *H. pylori* and *L. reuteri*, depend on the lipid compositions of bacteria. In fact, Gram-negative EVs that bleb from the external cell wall were enriched with different lipids and transport lipid components with a net negative charge derived from their native parental cell; furthermore, these membrane components can be enriched with nucleic acids, protein components and fatty acids which modify the resulting surface membrane charge of EVs [15]. Average sizes, size distribution and Z-potential were different in planktonic and biofilm whole samples before the EV isolations. These differences depended on the interaction between original bacterial cells and EVs, which were still fused in the samples before purification and generated broadly distributed particles with different sizes, and a decreased net negative surface charge due to the partial fusion of vesicles.

The purpose of the present study relies on an innovative, fast and simplified method, particularly reproducible, that, being already validated on eukaryotic-derived vesicles, was here tested, for the first time, for microbiological applications. Moreover, the detection of the eDNA-associated pEVs and bEVs represents a novel and valuable tool to better understand the role of eDNA delivered by these structures. However, we must not underestimate the application of PFC in the clinical field where it will be possible to detect and enumerate bacterial EVs in biological samples of infected patients.

## 4. Materials and Methods

### 4.1. Bacterial Strains and Media

*Lactobacillus reuteri* DSM 17938 [42], BioGaia AB, Stockholm, Sweden], and *Helicobacter pylori* ATCC 43629, were used for this study. The choice of these two microorganisms linked to the fact that in previous works we have demonstrated that they produce vesicles containing DNA, which are released both from the planktonic and the biofilm phenotypes [15,16]. *L. reuteri* DSM 17938 was spread on deMan, Rogosa, Sharpe Agar (MRS) (Oxoid Limited, Hampshire, UK), and incubated at 37 °C for 24 h in an anaerobic atmosphere (Anaerogen Pak Jar, Oxoid Ltd., Basingstoke, UK), while *H. pylori* ATCC43629 was plated on Chocolate Agar (Oxoid Ltd.) supplemented with 1% (*v*/*v*) of IsoVitaleX (Becton Dickinson, Franklin Lakes, NewJersey, NJ, USA) and 10% (*v*/*v*) of defibrinated horse sterile blood (Oxoid Ltd.), and finally incubated at 37 °C for 3 days in a microaerophilic atmosphere (Campy PakJar; Oxoid Ltd.).

### 4.2. Biofilm Formation Assay and Bacterial Vesicles Isolation

*H. pylori* ATCC 43629 and *L. reuteri* DSM 17938 biofilm development was carried out as previously reported [15,16]. The formation of biofilms was assessed by Syto 9 staining and Fluorescence Leica 4000 DM Microscopy analysis (Leica Microsystems, Wetzlar, Germany). Biofilms were rinsed with PBS, scraped and re-suspended in PBS for one more time. Four samples for both planktonic and biofilm phenotypes containing cells plus extracellular vesicles were analyzed by Flow Cytometry. The isolation of EVs from *H. pylori* and *L. reuteri* was carried out for both biofilm and planktonic phenotypes, as previously reported [15,16]. The resulting pellets were then re-suspended in 200 μL PBS and stored at 4 °C until the analysis. All the samples were treated with 1 U/µL DNase I (Sigma Aldrich, St. Louis, Missouri,) to avoid possible EV aggregations due to present eDNA [15]. DNase I-treated samples (whole samples consisting of cells plus pEVs or bEVs and isolated pEVs and bEVs) were subsequently analyzed by Flow Cytometry.

### 4.3. Flow Cytometry Analysis of EVs

Samples were stained as already reported [43]. Briefly, 100 μL of EV suspension/sample, obtained as above-described, was labeled with 4 μM PKH26, a probe for general cell membrane labeling (Sigma-Aldrich, St. Louis, Missouri, USA), 5 μL of Pico Green (Molecular Probes) for the staining of the dsDNA (internal dsDNA and/or external dsDNA-EVs associated), and 0.5 μL of lipophilic cationic dye (LCD, BD Biosciences—Catalogue, #626267, Custom Kit). Using the PicoGreen^®^ dsDNA quantitation assay (ThermoFisher, Waltham, Massachusetts, USA), we selectively detected the dsDNA in the presence of ssDNA, RNA, and free nucleotides, as reported by the manufacturer’s instructions. After 45 min of incubation at room temperature, 200 μL of PBS was added to the samples, and 1 × 10^5^ events/sample were acquired by flow cytometry (FACSVerse, BD Biosciences) by triggering on the channel in which LCD emits (APC Channel). Amplifier settings for forward scatter (FSC) and side scatter (SSC) as well as for any fluorescence channel were set in logarithmic mode, and all parameters were visualized in respect to the relative height (H) signal. As already published for eukaryotic-derived vesicles, EV morphology was confirmed by running Megamix Plus SSC and FSC beads (Biocytex, Marseille, France) at the same photomultiplier (PMT) voltages used for EV detection [22].

Each reagent was titrated under assay conditions; dilutions were established based on achieving the highest signal (mean fluorescence intensity, MFI) for the positive population and the lowest signal for the negative population, representing the optimal signal to noise ratio, and stain indexes were calculated [23]. Instrument performances and data reproducibility were implemented and checked by the Cytometer Setup & Tracking Module (BD Biosciences). In order to evaluate non-specific fluorescence, Fluorescence Minus One (FMO) controls were used [44,45]. The compensation was assessed using single-stained fluorescent samples. Data were analyzed using FACSDiva v 6.1.3 (BD), FACSuite v 1.0.5 (BD) and FlowJo v 8.8.6 (TreeStar, Ashland, OR, USA) software. EV numbers were obtained by volumetric count.

Colony Forming Unit and EV counts were carried out after 24 h of incubation for *L. reuteri* and after 48 h of incubation for *H. pylori* of both planktonic and biofilm phenotypes. The EVs/Cells Ratios were calculated (Table S1).

### 4.4. Physical—Chemical Characterization of pEVs and bEVs

The whole samples comprised planktonic or biofilm bacterial cells plus pEVs or bEVs as well as isolated pEVs and bEVs underwent a physicochemical characterization, using Dynamic Light Scattering (DLS), as previously reported [39,46]. The analysis was performed using a ZetasizerNanoZS (Malvern Instruments Ltd., Worchestershire, UK). DLS was set up according to the following parameters: 4.5 mW laser diode operating at 670 nm as a light source, and backscattering angle at 173°. A third-order cumulative fitting autocorrelation function was applied to measure the average sizes and the size distribution of EVs. The analysis was performed according to the following instrument setup: real refractive index of 1.59, imaginary refractive index of 0.0, medium refractive index of 1.330, medium viscosity of 1.0 mPa × s, and medium dielectric constant of 80.4, as previously reported [47,48]. To avoid artifacts due to the multiscattering phenomenon, samples were suitably diluted with isotonic RNase-free water before the analysis and pre-filtered with a 0.22 μm polypropylene membrane filter (Whatman Inc., Clifton, NJ, USA). Results are shown as an average ± standard deviation from 10 independent replicates. Surface charge or *Z*-potential of EVs was also tested by DLS. The *Z*-potential of EVs was calculated as a function of the electrophoretic mobility. The Doppler laser anemometry theory was applied to measure the *Z*-potential of EVs. A Smoluchowsky constant F (Ka) of 1.5 was applied during the analysis. The Doppler laser anemometry used to measure the *Z*-potential consisted of a He/Ne laser Doppler anemometry (633 nm) with a nominal power of 5.0 mW. Results were reported as the average ± standard deviation of 10 independent replicates.

### 4.5. Statistical Analysis

Statistical analyses were performed using GraphPad Prism ver. 5 (GraphPad Software Inc., La Jolla, Ca, USA). Results were represented as the average ± standard deviation (S.D.) or standard error of the mean (S.E.M.). The statistical analysis of data was performed using the t-test; while their statistical significance was set at *p* ≤ 0.05.

## Figures and Tables

**Table 1:** Physicochemical characterization of planktonic (p) and biofilm (b) supernatants and Membranes Vesicles (EVs) collected from *H. pylori* and *L. reuteri*. Supernatant contains a mixture of bacterial cells and EVs blebbing from *H. pylori* and *L. reuteri* respectively. EVs are the isolated forms derived from planktonic and biofilm phenotypes of the two bacterial species. The analysis is the average of ten independent replicates ± standard deviation.

Sample	PDI ^1^	*Z*-Potential (mV)	Electrophoresis Mobility (µm cm/Vs)
p@*H. pylori*	0.81 ± 0.3	−4.6 ± 0.3	−0.36 ± 0.02
b@*H. pylori*	0.85 ± 0.1	−8.37 ± 0.9	−0.67 ± 0.07
p@*L. reuteri*	0.26 ± 0.02	−13.1 ± 0.7	−1.02 ± 0.05
b@*L. reuteri*	0.21 ± 0.06	−8.38 ± 0.9	−0.65 ± 0.07
pEVs@*H. pylori*	0.22 ± 0.04	−21.9 ± 0.7	−1.72 ± 0.05
bEVs@*H. pylori*	0.77 ± 0.25	−17.9 ± 2.9	−1.4 ± 0.23
pEVs@*L. reuteri*	0.61 ± 0.02	−36.6 ± 0.4	−2.9 ± 0.02
bEVs@*L. reuteri*	0.75 ± 0.21	−24.6 ± 2.2	−1.9 ± 0.17

^1^ PDI = Polidispersity index.

## Supplementary Materials

Supplementary materials can be found at https://www.mdpi.com/1422-0067/20/21/5307/s1.
