# Supplementary material for: Detection and Quantification of eDNA-Associated Bacterial Membrane Vesicles by Flow Cytometry
Source: Int J Mol Sci. 2019 Oct 25;20(21):5307. doi: 10.3390/ijms20215307 (PMC6862651; doi:10.3390/ijms20215307)

**Supplementary Table 1**. EVs/Cells Ratios.		
	**Ratio EVs/Cells Biofilm**	**Ratio EVs/Cells Planktonic**
L. reuteri	1.02	3.82
H. pylori	1.10	8.17
Colony Forming Unit and EV counts were carried out after 24 hours of incubation for *L. reuteri* and after 48 hours of incubation for *H. pylori* of both planktonic and biofilm phenotypes.		

Supplementary Figure 1

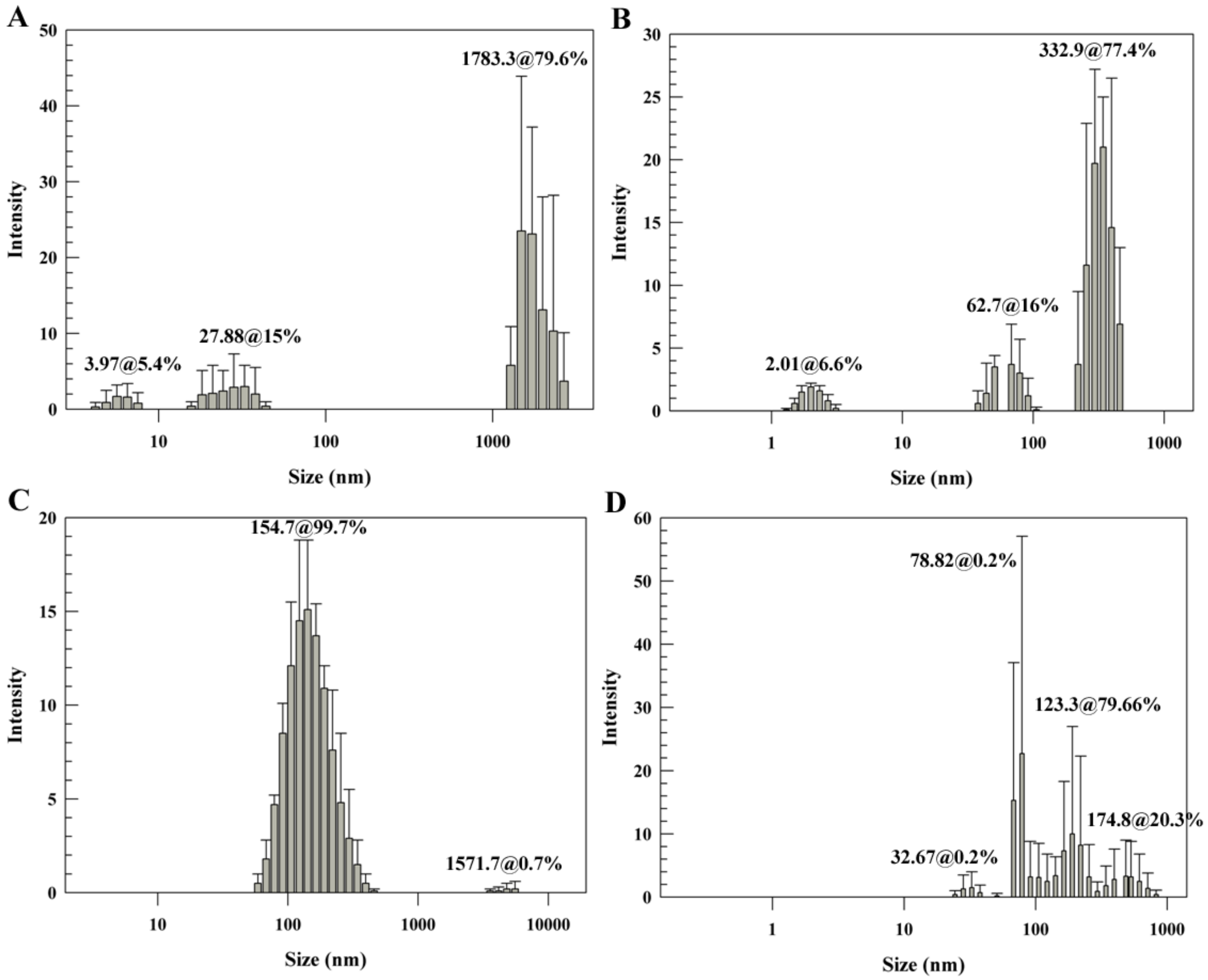

Figure S1 (DLS H. pylori panel). Physicochemical of MVs isolated from *H. pylori*. DLS analysis were the intensity percentage of average particles sizes of: planktonic (A) and biofil (B) mixture of membranes vesicles of *H. pylori* and cells before their isolation and purification; planktonic membrane vesicles or pMVs (C) and biofilm membrane vesicles or bMVs (D). Particles size had a monomodal distribution for pMVs (C) and a bimodal distribution for bMVs (D).

Supplementary Figure 2

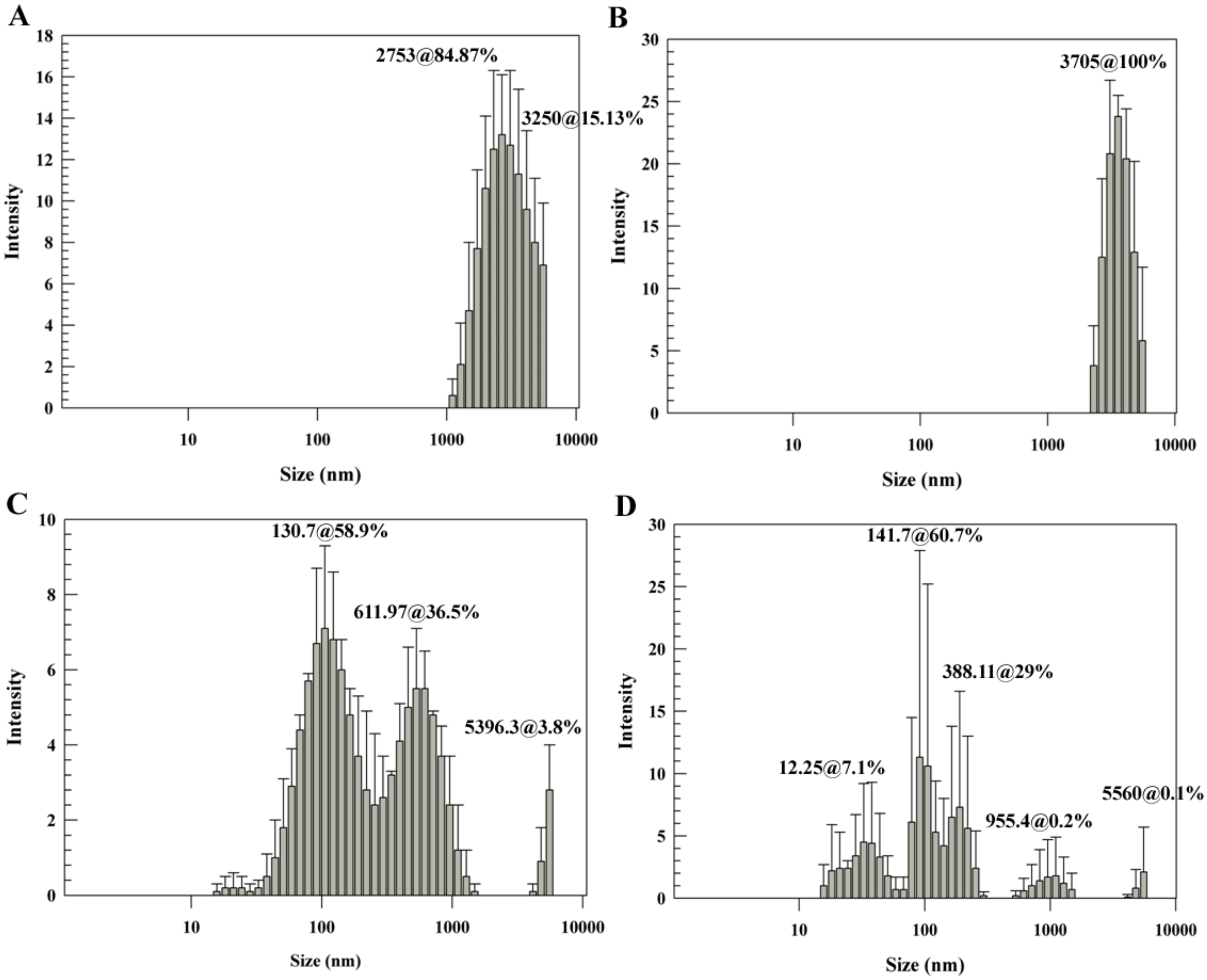

Supplement: Supplementary file 1 [file ijms-20-05307-s001.pdf]
